# Simultaneous production of alpha and beta amylase enzymes using separate gene bearing recombinant vectors in the same Escherichia coli cells

**DOI:** 10.3906/biy-2001-71

**Published:** 2020-08-19

**Authors:** Dilek ÖZCAN, Hikmet Murat SİPAHİOĞLU

**Affiliations:** 1 Department of Plant Protection, Faculty of Agriculture, Van Yüzüncü Yıl University, Van Turkey; 2 Department of Plant Protection, Faculty of Agriculture, Malatya Turgut Özal University, Malatya Turkey

**Keywords:** Alpha amylase, beta amylase, dual gene expression, purification of recombinant enzyme

## Abstract

The present study describes the simultaneous expression of thermostable industrial alpha (α) and beta (β) amylase enzymes that have been used widely in starch industry. Genomic DNA of *Bacillus stearothermophilus *DSM 22 strain for α amylase and, *Thermoanaerobacterium (Clostridium) thermosulfurogenes *DSM 2229 strain for β amylase were used as gene sources. Both genes were ligated into pETDuet-1 expression vector separately and resulting recombinant vectors were transformed into *Escherichia coli *BL21 competent cells by electroporation. The cells were first transformed by pETDuet-1/ αAmy recombinant plasmid, then the competent cells carrying this plasmid were prepared for the transformation of pETDuet-1/ βAmy plasmid. Enzymatic activities of bacterial colonies were detected on LB agar staining with iodide. Both enzymes were more produced by IPTG induction in BL21 cells and were purified using Ni-NTA agarose column. SDS-PAGE and western blot analyses showed that the molecular weight of purified α and β amylase to be approximately 60 kDa and 55kDa, respectively. The concentration of the purified α and β amylase were calculated as 4.59 μg/mL and 3.17 μg/mL with IPTG as an inducer in LB medium. The present study proposes a novel and efficient method for the production of thermostable α and β amylases at the same *E coli* cells containing separate engineered plasmid vectors.

## 1. Introduction

Amylases are among the most valuable commercial enzymes that commonly used in food, feed, paper, textile, leather, and detergent industries (Gözükara, 2009). These enzymes are widely distributed and constitutes the 25% of the world enzyme market. They have been considered to be one of the most important industrial enzymes. These enzymes can be obtained from various sources (i.e. plants, animals and microorganisms), however, because of their broad range of industrial applications and stability the microbial origins are generally considered the most suitable for industrial requirements (Reddy et al., 2003; Rajagopalan et al., 2008; Zafar et al., 2019). 

The obtaining of thermostable amylase enzymes from hyperthermophilic microorganisms from the culture involved many difficulties for industrial applications. This problem is thought to be solved by transferring the gene encoding the amylolytic enzyme from thermophilic microorganisms to mesophilic microorganisms. It has been found that the amount of these enzymes greatly increased, additionally that these thermostable enzymes produced in mesophilic cells generally exhibit no or very low activity at temperatures below the growth conditions of the organisms. Due to their overall natural features, the thermal denaturation of host cell culture ensures separation from mesophilic enzymes resulting high level and stable enzyme that suitable for industrial processes. Moreover, these enzymes are also resistant to proteolysis of the host cell (Bertoldo and Antranikian, 2002).

Alpha amylase enzyme, is the first enzyme used commercially (Radley, 1976). Alpha amylase, an endoenzyme, splits starch polymers (chain) into macromolecular dextrins and maltose by hydrolyzing α-1,4 bonds at random points. With its effect on amylose, viscosity decreases quickly, and starch’s property of rendering blue color with iodine disappears. The effect of this enzyme on the amylopectin molecule is similar to amylase, however, in this molecule, fragmentation does not take place in the α-1,6 bonds with branching (Reed, 1966). The field of application of thermostable *Bacillus* α amylase has been greatly extended and diversified. It has been used in paper industry to liquefy the starch, to produce glucose and fructose syrups and glue and in the fermentation of alcohol (Kıran et al., 2005).

Beta amylase, an exoenzyme, enables the formation of maltose units with two glucose molecules starting from the nonactive lead of chain by watering α-1,4 link of starch molecule. Enzyme does not hydrolyze amylose and amylopectin at random points but it follows a regular order (Ertugay, 2010). The most important feature of β amylase enzyme is that it is not activated at high temperatures. It was determined that *Clostridium thermosulfurogenes* produce a thermo-amylase enzyme β. Βeta amylase enzymes are used in maltose production and food and beverage industry. The resulting high purity maltose syrup is used in production of jam, confectionery, bread and beer, also as a sweetener in food (Tatar, 2007).

Like other polymers, starch molecules need combination of amylase enzymes to be completely hydrolyzed, and when α and β amylase enzymes are used together, they split starch better compared to their separate use (Haki and Rakshit, 2003; Ertugay, 2010). Simultaneous expression of two or more enzymes in one host has the advantages of avoiding repeated fermentations, reduces the extracting and purifying works, and improves the cost-effectiveness of the processes (He et al., 2014). Therefore, α and β amylase enzymes are used together in food, textile, detergent, and adhesive industries (Liu et al., 2003). In this study, molecular cloning and expression of α and β amylase genes were investigated with a special emphasis on recovery and partial molecular characterization of purified α and β amylases. 

## 2. Materials and methods

### 2.1. Materials

The genomic DNA of *Bacillus stearothermophilus (Geobacillus stearothermophilus*) DSM 22 strain and *Thermoanaerobacterium thermosulfurogenes (Clostridium thermosulfurogenes)* DSM 2229 strain were commercially obtained from Leibniz Institute DSMZ firm (German Collection of Microorganisms and Cell Cultures, Braunschweig, Germany) for α amylase (α Amy) and β amylase (β Amy) enzymes and genes, respectively. 

### 2.2. Primer design

For the PCR amplification of full-length α Amy and β Amy genes, primers (A-*Sac*I-F-5’-*AAAA*GAGCTCgGTGCTAACGTTTC-3’, A-*Hind*III-R-5’-* AAAA*AAGCTTTCAAGGCCATG-3’) (B-*BamH*I-F-5’-*AAAA*GGATCC*g*ATGATTGGAGCTTTTAAAAG-3’, B-*Xho*I-*R-*5’-*AAAA*CTCGAGTTAATTTTGCCATGTAATGGTG-3’) were designed using published sequence from the genomic database of *B. stearothermophilus* and *T. thermosulfurogenes* (Access number for α Amy gene: M57457 and for β Amy gene: M22471). The primers were compatible with the endonuclease restriction sites (*Sac*I, *Hind*III, *BamH*I, and* Xho*I) of pETDuet-1 expression vector (Novagen, Merck KGaA, Darmstadt, Germany). To each primer, four additional unrelated nucleotides (shown in italics) were added at their 5’end. For the amplification of both genes, a final volume of 50 µL PCR mixture contained: 2 µL of DNA, 5 µL of 10× reaction buffer (200mM Tris–HCl pH: 8.4, 500mM KCl), 3 µL of MgCl2 (25 mM), 1 µL of dNTPs (10mM each), 1 µL of each primer (100 pmol), 0.4 µL of Go *Taq* G2 Hot Start DNA polymerase and 36.6 µL of DNase free sterile water. The complete α Amy gene was amplified by PCR with the following thermal cycling scheme: 2 min at 95 °C, 30 cycles of 1 min at 94 °C, 1.5 min at 55 °C, and 2 min at 72 °C followed by a final extension at 72 °C for 10 min. The complete β Amy gene was generated by the following thermal cycling scheme: 2 min at 94 °C, 30 cycles of 20 sec at 94 °C, 20 sec at 55 °C, and 2 min at 72 °C followed by a final extension at 72 °C for 3 min. The PCR products were separated on 1% agarose gel and recovered by gel extraction kit (Thermo GeneJET Gel Extraction Kit, Thermo Fisher Scientific Inc., Waltham, MA, USA).

### 2.3. Construction of plasmids for the production and purification of recombinant His-tagged α and β amylase

The amylase gene sequences were cloned into the *Sac*I and *Hind*I site (for α Amy gene) and *Bam*HI and *Xho*I site (for β Amy gene) of u1d2d vector containing 6xHis-Tag coding sequence according to the protocol recommended by the manufacturer. The recombinant vectors were then transformed into competent cells of* E. coli* BL21(DE3)pLysS cells by Gene Pulser® apparatus (Bio-Rad Laboratories, Inc., Hercules, CA, USA), respectively. Then, they were plated on a LB agar containing ampicillin (100mg/mL). Positive clones were identified by colony-PCR, sequence analysis or by restriction endonucleases. Positively identified clones were inoculated into 10 mL of LB broth containing ampicillin and were grown to OD600 0.6. The bacterial growth was induced by adding isopropyl β-D thiogalactopyranoside (IPTG) to a final concentration of 0.4 mM followed by an incubation with constant shaking at 37 °C for 18h. The total broth media were centrifuged and bacterial cells were pelleted and resuspended with water containing cOmplete*™ *Mini EDTA*-*free tablet to inhibit proteolytic activity of a broad range of proteases (Roche Diagnostics International AG, Rotkreuz, ZG, Switzerland). After adding 0.5 mL of TrisHCl (pH7.5), 1 mL of NP40 (% 10), 25 µL of MgCl2 (1M), 7 µL of 2-mercaptoethanol and 20 µL of DNase I (10 U/µL), the suspension was sonicated four times (3–5 s) in ice, then, the mixture was incubated at 4 °C for 45 min on a shaker. After adding 0.3g of NaCl (final concentration of 0.5 M) the protein extract was ultracentrifuged at 30.000 rpm for 30 min (at 4 °C). Recombinant His-tagged α Amy and β Amy proteins were purified by chromatography on a Ni2+-NTA agarose resin column. After washing the column with TL buffer (2.5 mL 1M Tris-HCl pH 7.5, 1.46g NaCl, 5 mL %10 NP40, 35 µL 2-mercaptoethanol, 1mL 1M imidazole pH 8.0) the proteins was eluted with TE buffer (50 % of imidazole and 50 % of TL buffer). The protein concentrations were measured by the Bradford method (Bradford, 1976) using bovine serum albumin as standard.

### 2.4. Preparation of competent cells and transformation

A single bacterial colony was picked up from the plate incubated at 37 °C for overnight and transferred into 10 mL of LB medium overnight at 37 °C with shaking. The culture than added into 1000 mL of LB medium for 4 to 5 h at 37 °C with shaking at 250 rpm until reach the OD600 0.6. The culture centrifuged two times with ice-cold centrifuge tubes. The pellet was resuspended with 4 mL of chilled 0.8 % glycerine and aliquoted 100 μL of each tube. Recombinant pETDuet-1/αAmy plasmid was transferred to competent cells. Bacterial cells bearing pEtDuet-1/αAmy plasmids were then served for the preparation of competent cell for transformation of β Amy containing recombinant plasmids. Transformation was made by electroporation using a Gene Pulser® apparatus (Bio-Rad Laboratories, Inc.).

### 2.5. SDS-PAGE and western blot analysis and starch–iodine assay for extracellular amylase production

Levels of protein expression of recombinant BL21/αAmy and BL21/βAmy were analyzed by 10% Sodium dodecyl sulfate polyacrylamide gel electrophoresis (SDS-PAGE) along with molecular weight markers. The purified enzymes were loaded on PAGE gel than stained by coomassie blue as described by Laemmli (1970). Western blot analysis were carried out essentially according to Sambrook et al. (1989) using semidry blotting system. Purified proteins were transferred to PVDF-plus membrane by electro-blotting. Monoclonal antibody recognizing the His-tag residue was used to capture α and β amylases. Molecular weight of enzymes were estimated from a calibration curve of Log10 molecular weight of standard proteins. The amylolytic activity of transformed bacteria was determined by the method described by Cowan (1991), using SW-10 medium supplemented with 0.5% (w/v) soluble starch. The plates were than filled with 0.3 % I2 and 0.6 % KI solution and incubated at 37 °C for one week to observe a clear zone around the bacterial growth.

## 3. Results

### 3.1. Cloning and expression of thermostable α Amy and β Amy genes

**Figure 1:**
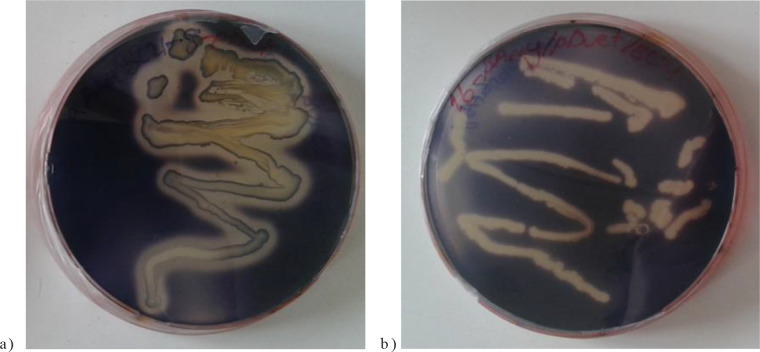
Testing of recombinant BL21/αAmy (A) and BL21/βAmy (B) bacteria with iodine staining developed in environment containing starch. Clear zones along zigzags indicate starch hydrolysis.

A full length α Amy and β Amy gene fragments of 1699 bp and 1675 bp were amplified from genomic DNA of *B. stearothermophilus *DSM 22 strain and *T. thermosulfurogenes *DSM 2229 strain, respectively, using gene specific primers. The cloning of α Amy and β Amy genes into pEtDuet-1 expression vector resulted the secretion of His-tagged recombinant enzymes into the periplasmic space and subsequently into culture medium. After induction with IPTG, the both recombinant enzymes were efficiently secreted. Under the standard assay conditions, the concentration of α and β amylase enzymes were measured as 4.59 µg/mL and 3.17 µg/mL, respectively. As seen in Figures 1A and 1B, the extracellular location of the enzymes are clearly evident form transformed bacteria that secrete α and β amylases (zigzags). The enzymes hydrolyzed the starch, creating a clear zone in which the iodine causes blue coloration of starchy medium. Iodine then degraded by secreted α and β amylase enzymes. 

### 3.2. Analysis of α and β amylases expressed in BL21/αAmy bacteria by SDS-PAGE

**Figure 2:**
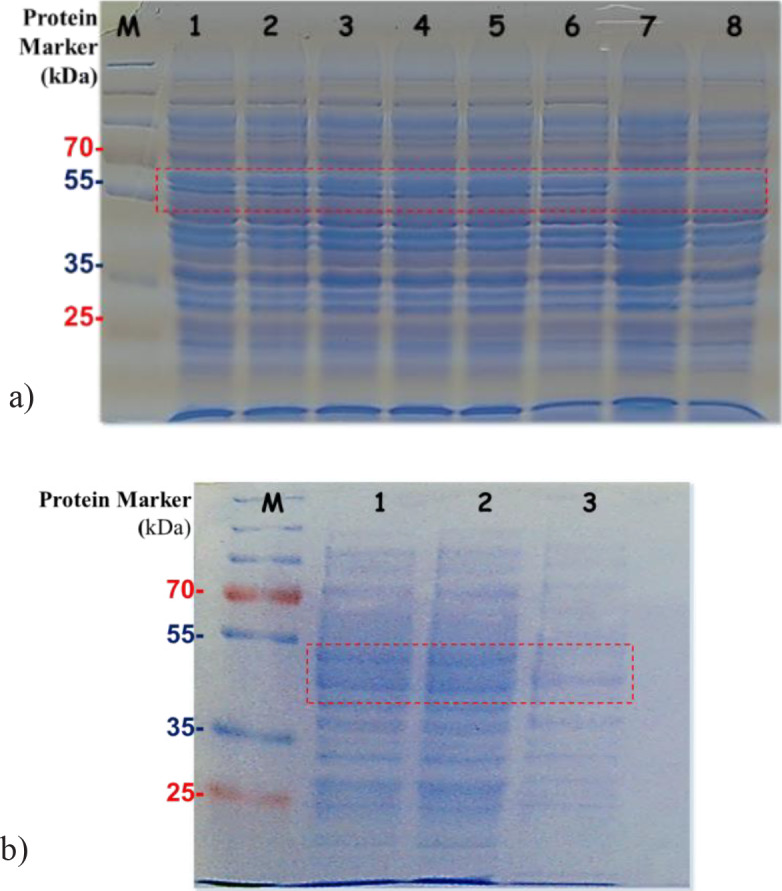
10% SDS-PAGE analysis of purified α (A) and β (B) amylase. Panel (A): Lane 1, 2, and 3 IPTG induced bacterial cells, Lane 4, 5, and 6 uninduced transformed cells, Lane 7 and 8 untransformed control cells. Panel (B): Lane 1 and 2 induced cells, Lane 3 untransformed control cells. M: Prestained protein molecular weight markers.

The α Amy and β Amy genes were under control of the T7 promoter in pEtDuet-1 expression vector. For the induction of high expression, isopropyl β-D-1-thiogalactopyranoside (IPTG) was used. The induced bacterial cells were used for affinity purification on Ni-NTA agarose. Levels of protein expression of recombinant BL21/αAmy and BL21/βAmy bacteria were analyzed by 10% SDS-PAGE (Figure 2). Recombinant α and β amylases showed a molecular mass of *ca*. 60 kDa and 55kD on SDS-PAGE, respectively. 

### 3.3. Western blot analysis

**Figure 3:**
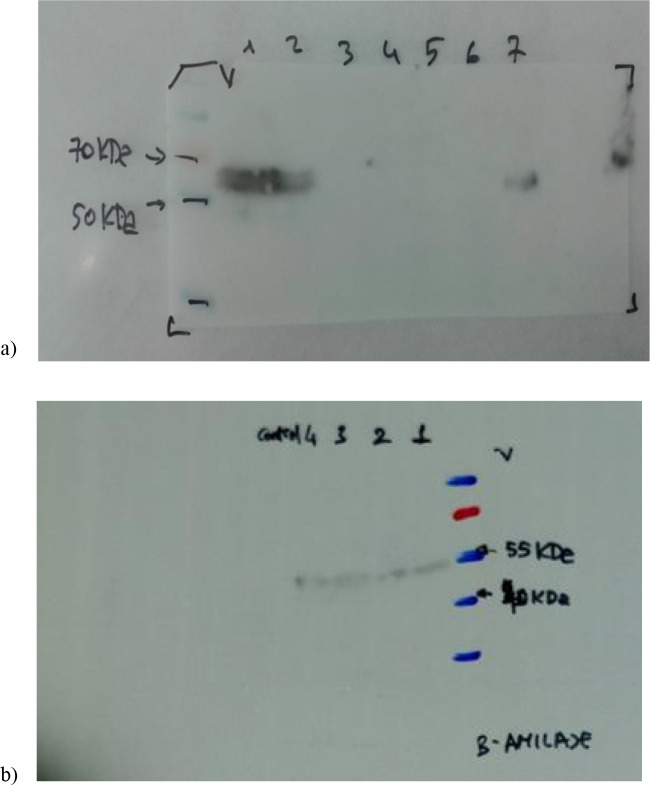
Western blot analysis of recombinant α (A) and β (B) amylase. Panel (A) Lanes 1 and 2 proteins of IPTG induced cells, Lane 3 protein of untransformed cells, Lanes 3, 4, and 5 uninduced cells, Lane 7 positive control. Panel (B) Lanes 1, 2, and 3 IPTG induced cells, Lane 4 positive control. M: Prestained protein molecular weight marker. Arrows indicate the molecular weight of α- and β-amylase at 60 and 55 kDa, respectively.

His-tag specific monoclonal antibodies captured α and β amylases resulting signals in western blot analyses. Signals were observed at a molecular weight of 60 and 55kDa confirming the presence of His-tagged recombinant proteins of α and β amylases, respectively (Figure 3). 

## 4. Discussion

As an expression host, *E. coli* has been widely used for the production of numerous industrial enzymes (Lin and Hsu, 1997; Lo et al., 2001; Shiina et al., 2007; Yamabhai et al., 2008). Generally, the enzymes are recovered from periplasmic space or cultivation medium (Shiina et al., 2007; Yamabhai et al., 2008). In the present study, transformants containing multiple copies of α and β amylase genes were obtained by electrotransformation of β amylase gene carrying plasmids to the α amylase gene bearing cells. The bacterial cells were then screened on plates for high-level expression of α and β amylase enzymes. In general, both enzymes have similar biochemical properties. Therefore, we firstly expressed α amylase in *E. coli* cells than these cells were served to prepare competent cells for the transformation of β amylase gene carrying plasmids in order to coexpress the two enzymes. Thus, the industrially advantageous of coexpression of both enzymes has been shown in the same host. The simultaneous expression of two or more enzymes in the same host has been reported by several researchers (Su et al., 1993; Li et al., 2011; He et al., 2014). He et al. (2014) demonstrated a significant improvement in amylase activity through coexpressing RpGla with RpAmy in *Pichia pastoris*. 

The correlation between starch hydrolysis zone and the relative enzyme expression rates has been shown in Figure 1. According to the hydrolysis result, starch digestion assay indicated that α and β amylases rapidly hydrolyzed the starch and created a clear zone on starch containing medium. The larger clear zones may be due to the high copy number of α amylase bearing plasmids and high level expression of amylases in *E. coli. *This finding coincides with the literature reported by Su et al. (1993). In the similar experiment carried out in* Zymomonas mobilis*, the larger zones of clearing produced by an enzyme indicate a high level of enzyme production. 

Based on western blot analysis, the purified recombinant α and β amylases showed two single band corresponding to MW 60 kDa and 55 kDA, respectively. The analysis confirmed that the both fusion proteins were correctly expressed. Molecular analysis showed that both proteins have molecular weights consistent with their monomeric native structure. In previous studies carried out by Gandhi et al. (2015) and Özcan et al. (2001), the molecular mass of the recombinant α amylase from *Geobacillus stearothermophilus* was calculated as 59 kDa and 65 kDa from *Bacillus subtilis*, respectively. The molecular mass of the recombinant β amylase from barley calculated as 60 kDa by Ziegler (1999). There has been a high variation in molecular mass of α and β amylases which varies in the range of 10–210 kDa (Gupta et al. u1d30).

We purified two different recombinant amylases which cosecreted from same bacterial cells in varying amounts. However, α amylase molecule having higher molecular weight was seen predominant over the β amylase. The concentration of purified α amylase was approximately 20% higher than β amylase. This may be due to low copy numbers of β amylase bearing plasmids in *E. coli* cells. In a previous study, Castro et al. (1992) reported a simultaneous production of bacterial α and β amylases with a continuous culture technique in separate hosts. However, novel approaches are needed to obtain high level of expression in the same host from the cloned genes. In the present study, we developed an efficient expression system for the heterologous expression of recombinant α and β amylase genes using T7 promoter-based pET vector at the same cells. *E. coli* BL21(DE3)pLysS cells carrying αand β amylase bearing plasmids simultaneously produces recombinant α and β amylase when growing in LB culture medium. The enzyme concentrations were comparable with those reported enzymes produced alone. The present study offer a promising method to prepare a high yield of α and β amylase enzymes. To our knowledge, the present study is the first report on the simultaneous expressing of α and β amylase enzymes using separate expression vectors in the same host. 

## Acknowledgments

This study was supported by research grant from Van Yüzüncü Yıl University Scientific Research Projects Department (Project no: BAP 2013-FBE-YL033).
